# Evaluating the Applications of Dendritic Neuron Model with Metaheuristic Optimization Algorithms for Crude-Oil-Production Forecasting

**DOI:** 10.3390/e24111674

**Published:** 2022-11-17

**Authors:** Mohammed A. A. Al-qaness, Ahmed A. Ewees, Laith Abualigah, Ayman Mutahar AlRassas, Hung Vo Thanh, Mohamed Abd Elaziz

**Affiliations:** 1 College of Physics and Electronic Information Engineering, Zhejiang Normal University, Jinhua 321004, China; 2 Department of Computer, Damietta University, Damietta 34517, Egypt; 3 Faculty of Information Technology, Al-Ahliyya Amman University, Amman 19328, Jordan; 4 Faculty of Information Technology, Middle East University, Amman 11831, Jordan; 5 Faculty of Information Technology, Applied Science Private University, Amman 11931, Jordan; 6 School of Computer Sciences, Universiti Sains Malaysia, Pulau Pinang 11800, Malaysia; 7 School of Petroleum Engineering, China University of Petroleum (East China), Qingdao 266580, China; 8 Laboratory for Computational Mechanics, Institute for Computational Science and Artificial Intelligence, Van Lang University, Ho Chi Minh City 700000, Vietnam; 9 Faculty of Mechanical-Electrical and Computer Engineering, Van Lang University, Ho Chi Minh City 700000, Vietnam; 10 Department of Mathematics, Faculty of Science, Zagazig University, Zagazig 44519, Egypt; 11 Faculty of Computer Science & Engineering, Galala University, Suze 435611, Egypt; 12 Artificial Intelligence Research Center (AIRC), Ajman University, Ajman 346, United Arab Emirates; 13 Department of Electrical and Computer Engineering, Lebanese American University, Byblos 4307, Lebanon

**Keywords:** dendritic neural regression (DNR), particle swarm optimization, metaheuristic, time-series, forecasting, oil production

## Abstract

The forecasting and prediction of crude oil are necessary in enabling governments to compile their economic plans. Artificial neural networks (ANN) have been widely used in different forecasting and prediction applications, including in the oil industry. The dendritic neural regression (DNR) model is an ANNs that has showed promising performance in time-series prediction. The DNR has the capability to deal with the nonlinear characteristics of historical data for time-series forecasting applications. However, it faces certain limitations in training and configuring its parameters. To this end, we utilized the power of metaheuristic optimization algorithms to boost the training process and optimize its parameters. A comprehensive evaluation is presented in this study with six MH optimization algorithms used for this purpose: whale optimization algorithm (WOA), particle swarm optimization algorithm (PSO), genetic algorithm (GA), sine–cosine algorithm (SCA), differential evolution (DE), and harmony search algorithm (HS). We used oil-production datasets for historical records of crude oil production from seven real-world oilfields (from Tahe oilfields, in China), provided by a local partner. Extensive evaluation experiments were carried out using several performance measures to study the validity of the DNR with MH optimization methods in time-series applications. The findings of this study have confirmed the applicability of MH with DNR. The applications of MH methods improved the performance of the original DNR. We also concluded that the PSO and WOA achieved the best performance compared with other methods.

## 1. Introduction

Forecasting oil production is an urgent means for petroleum engineers and oil companies to form effective links between oil reservoir developments and their profitability [1,2,3]. To achieve this ultimate goal, two elements need to be accomplished. Firstly, a robust and reliable geostatistical reservoir model is required to attain precision [4]. The geostatistical reservoir model consists of various elements, including the structural model, the lithofacies model, and the petrophysical model; hence, generating an accurate geostatistical model is an important stage [5,6,7].

However, developing an accurate geostatistical model is challenging and cumbersome. Secondly, a dynamic reservoir modeling approach must be developed; this comprises various parameters, including PVT [8], testing wells [9], and production of wells [10]. The dynamic reservoir mainly depends on the quality of the history-matching performance [11]. However, the accuracy of history matching relies on the fit of the oil-production historical data, the quality and quantity of pressure data, and the accuracy of the geostatistical reservoir model. The history-matching mechanism is challenging and time-consuming. Hence, integrating geological models and dynamic models is the main step to ensure that we can perform oil-production forecasting [3,4,7].

In the petroleum industry, there are different traditional approaches that are engaged in predicting oil reservoir production, including decline curve analysis (DCA) [12] and numerical reservoir simulation (NRS) [13]. The aforementioned techniques have some advantages and disadvantages in terms of forecasting production. Decline curve analysis (DCA) is a set series for estimating long-term reserves and determining the estimated ultimate recovery (EUR), which has been performed effectively in oil and gas reservoirs for decades. The DCA entails fitting the historical-production data to an empirical equation and then extrapolating the production patterns. On the other hand, it is challenging to fit the historical production of oil wells. Even with good historical matching, there potential remains for calculating unreliable predictions because of tedious and unsteady production settings. NRS is touted as the optimal conventional method for predicting oil production by emulating and observing historical oil well production [14]. The success of achieving good performance using NRS is associated with the accuracy of the geological model and the quality of historical oil production. The NRS is a good means of predicting oil production; however, the NRS involves investing significant time in a trial-and-error development method. Furthermore, it is challenging to attain precision. Although the DCA and NRS have been widely implemented in many oil and gas fields, these conventional approaches still have certain limitations in terms of accuracy, difficulty, and time consumption. As a result, it is critical for developed new techniques that have the ability to develop forecasting models with high accuracy in a short time.

To address these issues in the petroleum industry, deep learning and machine learning have emerged as powerful techniques to tackle the aforementioned issue. Deep learning and machine learning have been involved in numerous studies of oil production. Recently, many researchers have investigated the capability of deep learning and machine learning to forecast oil production. Liu et al. [2] applied ensemble empirical mode decomposition (EEMD) based on long short-term memory (LSTM) to forecast oil production. Empirical findings revealed that the presented framework is efficient in providing adequate production forecasts. In [15], the authors applied a deep-gated recurrent unit (GRU) approach to forecast oil production. They demonstrated their model’s ability to capture the long-term dependencies of time-series data instead of using huge amounts of memory. The authors in [3] employed various machine learning techniques to develop models that forecast the oil well production in the Volve field. They revealed that the developed models have a high-prediction-output performance. In [7], the authors developed and optimized a new hybrid intelligence time-series model, namely the Aquila optimizer–adaptive neuro-fuzzy inference system (AO-ANFIS), to predict oil production. The output indicated that the developed model is a powerful time-series tool for forecasting oil production. Furthermore, in [4], the authors developed and optimized an artificial time-series to forecast oil production from two oilfields in different countries. The developed model consists of an adaptive neuro-fuzzy inference system, a slime mold algorithm (SMA), and opposition-based learning (OBL); thus, the developed model was named ANFIS-SMAOBL. The developed model demonstrated a strong ability to forecast oil production effectively. The authors of [1] developed advanced time-series forecasting (TSF) to predict oil production using deep LSTM (DLSTM). These authors emphasized the ability of deep learning to precisely forecast oil production.

Deep neural networks have been widely employed in time-series forecasting and prediction problems. The dendritic neural regression (DNR) is one of the promising neural network models that was adopted in time-series forecasting [16,17]. However, DNR faces specific challenges in the parameter configuration, which affects its performance. Inspired by the recent advances in metaheuristic (MH) optimization algorithms that are adopted in different engineering applications, in this paper, we study the applications of different MH optimization algorithms in optimizing DNR. The main idea is to test the performance of the DNR using the power of MH algorithms that are employed to optimize the parameters and to boost the forecasting performance. We selected several well-known optimization methods: a particle swarm optimization algorithm (PSO), a whale optimization algorithm (WOA), a genetic algorithm (GA), a sine–cosine algorithm (SCA), differential evolution (DE), and a harmony search algorithm (HS). We evaluate the optimized DNR model using real-world datasets of oil production collected from a real oilfield in China and provided by a local partner. We found that the applications of MH with DNR have certain impacts on the models’ forecasting performances.

## 2. Preliminaries

In this section, we present the preliminaries of the applied methods: DNR, whale optimization algorithm (WOA), particle swarm optimization algorithm (PSO), genetic algorithm (GA), sine–cosine algorithm (SCA), differential evolution (DE), and harmony search algorithm (HS).

### 2.1. Basics of the DNR Model

The basic DNR model generally consists of four layers. It is known that the first layer is called the synaptic layer. The main function of this layer is to receive the input data. After that, The received input data can then be passed by the defined activation function to the next layer. The next or the second layer is called the dendrite layer. This layer has branches that can be employed to send the input data into the next layer (the third layer), which is called the membrane layer. The main function of this layer is to integrate all received data that has passed through the previous layers; then, it delivers them into the next layer, the soma layer. The soma layer uses the defined sigmoid function to process the received data and to generate the outputs. The mathematical models of the above-mentioned steps are presented here.

(1) **Synaptic layer:**

The synaptic layer simulates the nervous system’s synaptic components [18,19]. Equation (1) is employed to process the input data received by this layer:

(1) $$D_{ij}=\frac{\omega_{i}}{1+e^{-a{\left(\frac{\omega_{i,j}x_{i}-\theta_{ij}}{\alpha_{i}}\right)}^{,}}}$$

where *x_i_* refers to the *i*th input data, and *D_ij_* indicates the values of *i*th synapse delivered to the *j*th dendritic branches. Moreover, ɑ represents a positive constant parameter. Additionally, *w_im_* and ɵ*_im_* refer to alterable parameters used for different tasks.

(2) **Dendrite layer:** The dendrite layer is used to aggregate the input data from the first layer. The input data have nonlinear relationships. They can play a vital role in neural information processing. This nonlinear relationship is represented by Equation (2)

(2) $$M_{j}=\prod_{i=1}^{I}y_{ij}$$

where $M_{j}$ indicates the output values of each *m*th dendritic branch.

(3)**Membrane layer:** The membrane layer can be used to aggregate the input data from the branches of the previous layer (dendrite layer). Then, a summation is applied to perform the integrated task, as represented by Equation (3):

(3) $$S=\sum_{j=1}^{J}\left(u_{j}{*M}_{j}\right)$$

where $u_{j}$ indicates the strength of dendritic branches and *S* refers to the input of the next layer, the soma layer. Generally, $u_{j}$ is set to 1 in DNM, and for DNR, the µm represents a variable parameter used in different processes by adjusting its values to be able to deal with regression problems [19].

(4) **Soma layer:** The Soma layer is the last layer, which uses the sigmoid function as an activated function. Additionally, the cell body could be fired if the membrane exceeds the threshold. Equation (4) presents the mathematical definition of this problem:

(4) $$R=\frac{1}{1+e^{-A(S-v)}}$$

where *R* is the output of the soma layer, whereas ɑ and *v* are positive constants.

### 2.2. Whale Optimization Algorithm (WOA)

The WOA was developed by Mirjalili [20] and was inspired by the unique wildlife strategic plan of humpback whales, which is known as the bubble-net feeding technique. As a result of this, Mirjalili suggested a new nature-enlightened algorithmic optimization technique, which is known as the WOA. This method simulates the behavior and attitude of humpback whales [20]. It is one of the most efficient optimization methods and has received widespread attention in recent years.

### 2.3. Particle Swarm Optimization Algorithm (PSO)

The PSO method is one of the earliest and most famous swarm intelligence optimization algorithms. It is based on the interaction and dispersal techniques of flocks of birds and is a stochastic optimization technique [21]. To begin the PSO investigation, a swarm of individuals, each representing a particle, is first created. These characters correspond to propose options towards the optimization task, as shown by the corresponding positions of these letters. For each particle, the speed at which it moves during the global search is also taken into consideration [22].

### 2.4. Genetic Algorithm (GA)

Many optimization issues can be overcome using an evolutionary algorithm such as a genetic algorithm (GA). During the optimization procedure, various types of genetic operators were used as the basis for a GA. In the beginning, the GA relied on a completely random group with likely solutions. Chromosomes serve as a visual representation of the latter. It can then be used to discover the ideal solution by applying genetic operators such as crossover, replication, and mutations. Simply replacing unfit members with fresh ones based on a fitness value which provides the optimal solution to also be maximized was performed during the focus on the improvement of GA. Individuals are selected to be parents by GA’s selection operator. Those individuals are also selected to serve as ancestors for future generations. Two individuals randomly share information during a crossover. During a mutation, the bits of a gene’s code can be changed at random. When the requirement for pausing is met, this cycle keeps going until a level of performance is reached that is good enough for the optimized task [23].

### 2.5. Sine–Cosine Algorithm (SCA)

The SCA is a population-based MH optimization method. It was inspired by sine and cosine functions in mathematics to address complex optimization tasks and problems.

The main idea is to initialize multiple initial random candidate agents. Those agents are required to fluctuate towards and away from the best agent (solution). This process depends on applying a mathematical model, relying on sine and cosine functions [24]. It showed competitive performance in recent years, as it was adopted in different applications and optimization problems [25].

### 2.6. Differential Evolution (DE)

Differential evolution (DE) is a type of method that was established by Storn and Price and is used for both evolutionary and global optimization purposes [26]. DE is indeed a branch of GA, and both of these algorithms utilize the same operators—crossover, mutation, and selection—albeit in somewhat different ways. Furthermore, mutations are the main search operators in DE, whereas selecting has been used to help guide the search to the most promising regions.

### 2.7. Harmony Search Algorithm (HS)

The HS can be defined as a music-based MH optimization method. The main inspiration of the HS method came from music harmony, in which some effort can be made to find harmony in music, which can be considered as a solution for optimization problems during the search process in [27,28].

### 2.8. Methodology

The DNR presented in this study was trained using several types of optimization techniques, namely the whale optimization algorithm (WOA), the particle swarm optimization algorithm (PSO), the genetic algorithm (GA), the sine–cosine algorithm (SCA), differential evolution (DE), and the harmony search algorithm (HS). Different optimization techniques were used to determine which was the most suitable for effectively training the DNR’s weights and threshold parameters. In this stage, the proposed model starts by determining all experiments’ parameters and preparing the used dataset. Then, the optimization technique searches for the best DNR parameters using the optimization technique. After that, the selected parameters can be applied to train the DNR. The obtained results are evaluated by Equation (5) to check the qualities of the new parameters.

(5) $$MSE=1/n\sum_{i=1}^{n}(b_{oi}-{\left(b_{ci}\right)}^{2})$$

where $b_{o}$ denotes the real data. $b_{c}$ denotes the output data. N denotes the data size. In this regard, the best parameters are selected considering the value of the MSE between the target and output data; namely, the smallest MSE is the best. These sequences work till reaching the stopping condition and the maximum number of fitness function evaluations. After finishing the training phase, the best parameters are used for the DNR to start the testing phase. The following pseudo-code (Algorithm 1) shows the sequence used in each algorithm to optimize the DNR model.
**Algorithm 1:** The MH-DNR pseudo-code1:Determine all used parameters.2:Randomly initialize the population.3:Compute the initial objective values for the population.4:Select the best solution.5:Initialize the iteration i=06:**while** (i < max iteration) **do**7:    Update the parameters of the optimization process, such as random and control parameters.8:    Update each solution using the conditions of the MH algorithm.9:    Pass the solution on to train the DNR model.10:    Calculate the objective value using the objective function.11:    Save the best value.12:    Increase i=i+113:**end while**14:Return the best DNR parameter.

## 3. Experimental Evaluation

### 3.1. Dataset

**Figure 1:**
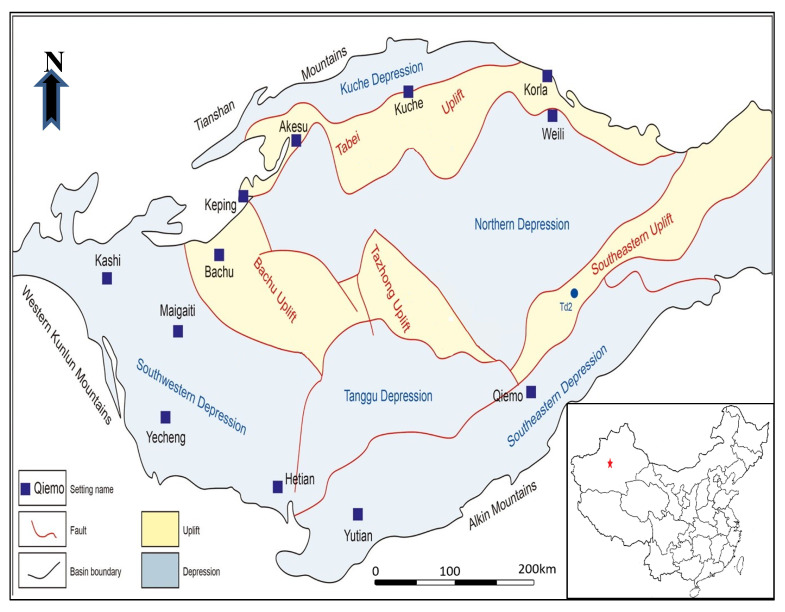
The study area: S3 oilfield, Tahe oilfield, Block 9, China.

We used real-world oil-production datasets of seven oilfields within the Tahe oilfield, China, provided by a local partner. Tarim basin is located in the Xinjiang region, and it consists of several oilfields, including the Tahe oilfield [7,29]. Tahe oilfield is situated in Luntai county, in Northwest China, and is considered the most productive oilfield in the Tarim basin [30]. Tahe oilfield consists of several oilfields, including the S3 oilfield, the block-6, and the block-9 oilfield. The S3 oilfield is located in the upper part of the Tahe oilfield, and it has roughly 28 wells, with a total area of 8.47 km${}^{2}$ [31]. Moreover, the S3 is characterized by good reservoir properties. Figure 1 shows the S3 oilfield location.

### 3.2. Results

In this section, the results of the proposed method, compared with other comparative methods, are presented in terms of various evaluation measures (i.e., root mean square deviation (RMSE), R-squared ($R^{2}$), mean squared error (MSE), and mean absolute error (MAE)). These measures are standard in this domain for evaluating the achieved results and for validating the algorithm performance compared with other existing methods. The proposed method is compared with other comparative methods, such as the whale optimization algorithm (WOA), the particle swarm optimizer (PSO), the genetic algorithm (GA), differential evolution (DE), the sine–cosine algorithm (SCA), and the harmony search (HS) optimizer. All experiments were applied over MATLAB 2014b using “MS Windows 10” with “Intel Corei7 CPU” and 8 GB of RAM.

Table 1 shows the results of the comparative methods in terms of RMSE measure. The RMSE represents the degree of dispersion of these residuals. In other words, it provides information on how tightly the data are clustered around the line of best fit. The DNR—modified using the PSO method—clearly obtained the best results in most tested cases, such as well numbers 1, 2, 3, 4, and 6, in terms of RMSE.

Table 2 shows the results of the comparative methods in terms of the R-squared ($R^{2}$) measure. R-squared is a quantitative metric that indicates how much of the variance for a dependent variable in linear regression is explained by one or more predictor factors. This measure ($R^{2}$) shows that the DNR modified using the WOA method (for tuning the forecasting model) obtained the best results in most tested cases. It is clear from Table 2 that the best results were achieved by the DNR modified using the WOA and PSO optimization methods.

Table 3 shows the results of the comparative methods in terms of the MSE measure. A fitted line’s MSE gauges how near it is to the data points. The value for each data point is squared by measuring the vertical distance between the point and the given input value on the curve fit. In terms of MSE measure, the modified DNR using the PSO method obtained three best results out of seven, followed by the traditional DNR, which obtained two best results out of seven. It is clear that the DNR-PSO method obtained the best forecasting performance compared with the other methods.

Table 4 shows the results of the comparative methods in terms of the MAE measure. MAE measures mistakes between paired observations, reflecting the same phenomena as that in the statistics. Analyses of expected data versus observed data, subsequent time versus initial time, and one measuring technique versus an alternate measurement technique, were alike to Y versus X comparisons. It is clear that, in Table 4, the performance of the modified DNR using PSO is the best, followed by the modified DNR using the WOA algorithm. Thus, according to the given results, we can conclude that both the PSO and the WOA optimization methods have significant impacts on the performance of the traditional DNR. They can be used with the DNR to improve the time-series forecasting problems, such as the crude-oil-production forecasting problem. They showed a good ability to deal with the forecasting of time-series data.

**Figure 2:**
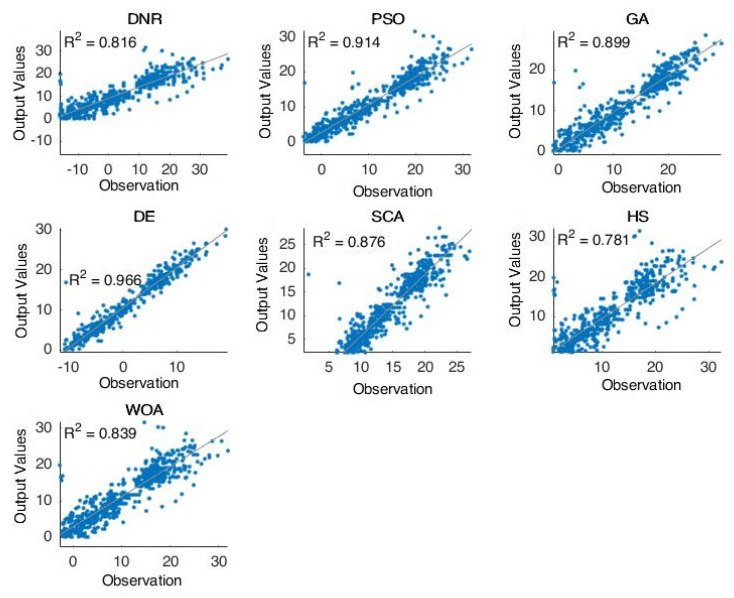
The results of well 4.

**Figure 3:**
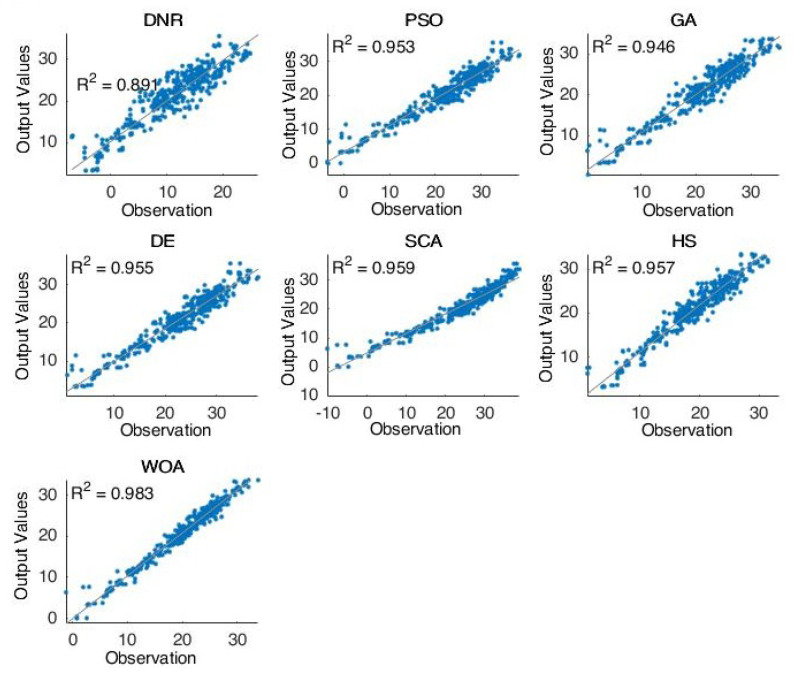
The results of well 5.

**Figure 4:**
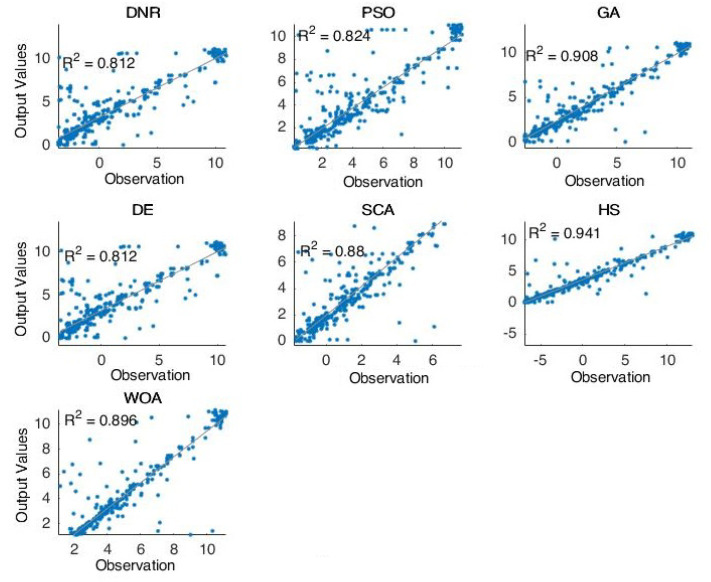
The results of well 7.

According to the visual results, the plots given in Figure 2, Figure 3 and Figure 4 show the results of the tested cases using various optimization methods. Figure 2 shows the forecasting results of the comparative methods for oil well number 4. It is clear that the optimization methods can forecast the targeted results. The PSO was shown to be the best-performing method, obtaining results which were near the actual values through the prediction process, followed by WOA and DE. These results proved the general performance of such hybrid optimization methods with DNR in solving difficult time-series problems.

Figure 3 shows the forecasting results of the comparative methods for oil well number 5. For the second case (well 5), we can see that the obtained results are very close to the best results. Furthermore, in this case, the PSO and WOA obtained the best forecasting results compared with the other methods. Figure 4 shows the forecasting results of the comparative methods for oil well number 7. For the last case (well 7)—one of the most complicated models in this research—the PSO and WOA were shown to be the best methods for solving this case. Moreover, the results proved the optimization technique’s excellent ability to handle completed problems.

**Figure 5:**
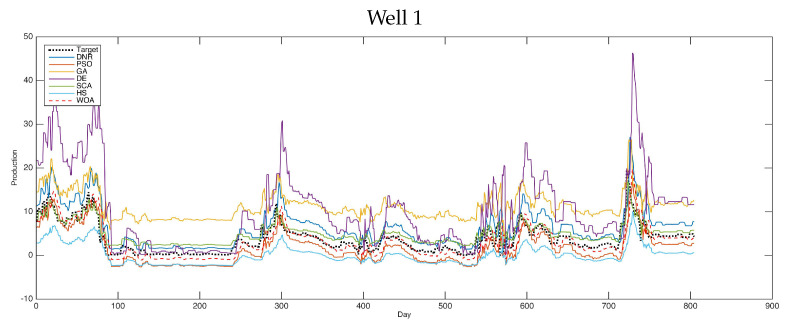

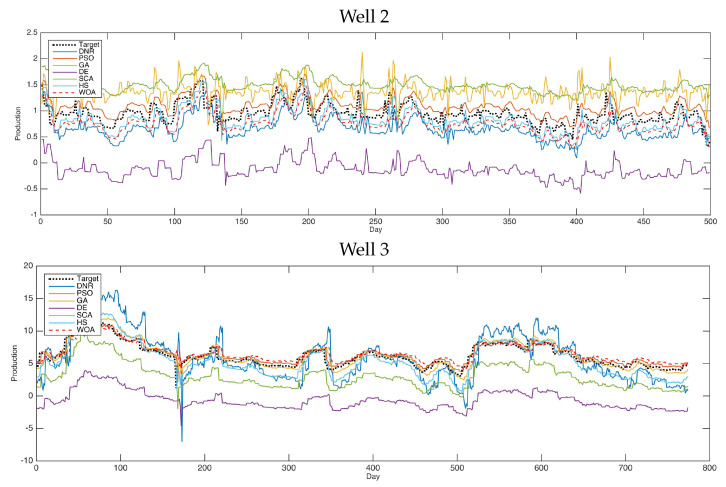
Forecasting results of all methods (i.e., well 1–well 3).

**Figure 6:**
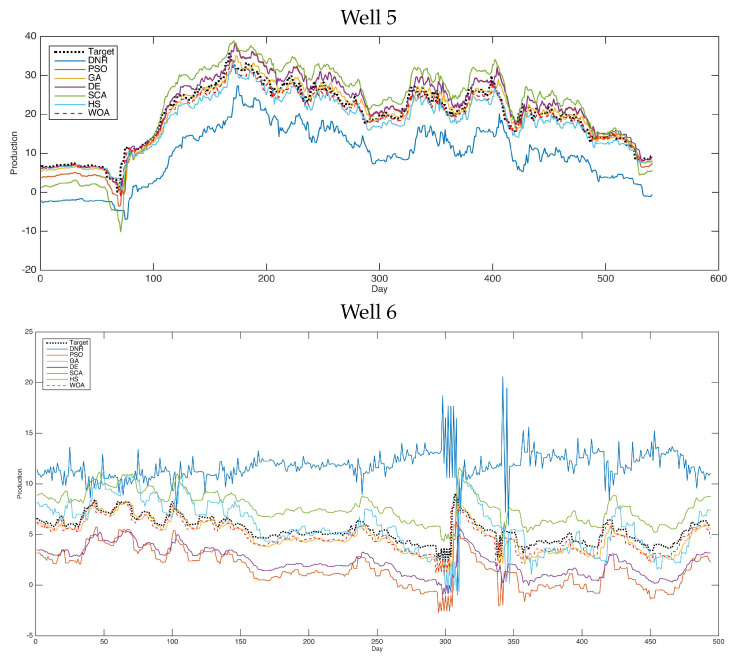

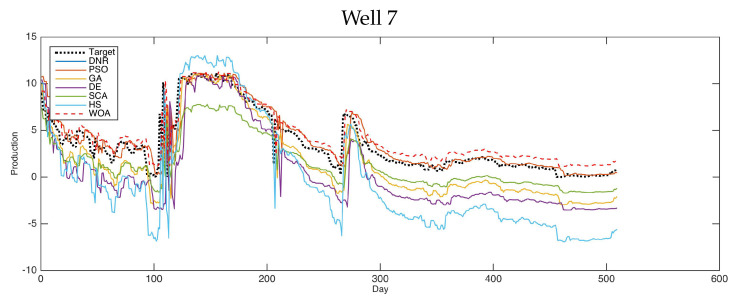
Forecasting results of all methods (i.e., well 4–well 7).

Additionally, Figure 5 and Figure 6 shows the forecasting results of six selected oil wells using various advanced optimization methods. As shown in these figures, the performances of the tested methods are clearly presented. We can see that the results obtained by PSO and WOA are close to the targeted results in these figures. This type of experiment is helpful in this field for determining the capabilities of the methods used in forecasting by displaying the resulting data and comparing it with the actual recorded data. Therefore, it is easy to judge here which methods are more robust and better for use in solving such problems. In general, improvement methods have proven themselves in dealing with such problems and obtain better results than the traditional methods used in this field.

### 3.3. Discussion

In this section, we discuss the achieved results. In our opinion, the applications of MH optimization algorithms have significant impacts on the forecasting performance of the DNR. As given in Table 1, Table 2, Table 3 and Table 4, the forecasting method using the PSO- and WOA-optimized DNR is the best approach for obtaining the most accurate forecasting results. As noticed from Table 1, the PSO-optimized DNR is promising for tuning the forecasting model, and it can lead to optimal forecasting results. Thus, the dendritic neural regression obtained better results in tuning its parameters with the support of the optimization method. From Table 2, we can see that the obtained results show the ability of the optimized DNR models to extract the most accurate outputs. This supports the modification to the proposed method used during the research process to find better solutions to solve such problems. It is clear that the WOA recorded the best $R^{2}$ results. Additionally, for the MSE indicator (as shown in Table 3, we can notice that the optimized DNR with PSO obtained the best performance, whereas the original DNR obtained the second-best performance. This confirms the capability of the PSO to boost the forecasting ability of the DNR model. Furthermore, the performance of the optimized DNR using PSO also recorded the best results. We note that the PSO outperformed other comparative optimization algorithms used to train and optimize the DNR.

Furthermore, Figure 2, Figure 3, Figure 4 and Figure 5 proved the superior ability of the PSO- and WOA-optimized DNR over the comparative methods in solving the time-series forecasting problem.

However, some other problems face individual MH optimization algorithms while searching for solutions, such as being stuck at local optima and convergence speed. These challenges sometimes have a terrible impact on the solution quality. Thus, the hybrid concept could solve the issue of improving an individual MH optimizer; for example, two MH algorithms could be combined to utilize both their advantages and to avoid their individual limitations. This concept could be utilized in future work for more complex forecasting problems using the DNR. In the current study, the application of an individual MH is sufficient for producing acceptable forecasting results with reduced computation complexity.

## 4. Conclusions

Oil-production forecasting is critical in making necessary plans for developing counties. Artificial neural networks have recently been utilized in many applications, including time-series forecasting and prediction. Dendritic neural regression (DNR) is an efficient ANN that has shown good performance in various applications, including forecasting applications. In this study, we presented a comprehensive evaluation of the applications of the metaheuristic optimization algorithms for optimizing the DNR model. It is known that the DNR can deal with the nonlinear characteristics of historical time-series data for prediction and forecasting applications. However, its parameter-configuration process faces some challenges and limitations. To this end, MH optimization methods can be employed to improve configuration and to enable a method to obtain the best solutions. In this study, we used six MH algorithms to optimize the DNR: the whale optimization algorithm (WOA), the particle swarm optimization algorithm (PSO), the genetic algorithm (GA), the sine–cosine algorithm (SCA), differential evolution (DE), and the harmony search algorithm (HS). We used real datasets for oil production from a real oilfield in China provided by a local partner. We implemented extensive evaluation using performance indicators, including RMSE, MAE, $R^{2}$, and MRAE. We found that the applications of MH significantly improve the prediction results. In short, the main contributions can be presented as follows:

- We present the first application for the DNR in the oil industry. This is the first time that DNR has been used to forecast oil production.
- We present six optimized DNR models using the advances of MH and swarm intelligence algorithms. The main idea was to optimize DNR parameters using the selected MH optimization algorithms to boost the forecasting capability of the DNR.
- We implement extensive evaluation experiments with real-world oil-production datasets that contain different oil wells’ historical production records to evaluate the six modified DNR models and compare their outcomes.

However, using an individual MH optimization algorithm to train the DNR has some limitations, especially during the search process. Sometimes, an individual MH algorithm may be stuck at local optima while searching for the optimal solution. Thus, hybrid MH methods could be employed in future work to optimize DNR models, avoiding those limitations. In future work, an optimized DNR could be applied to other applications, such as air pollution prediction and analysis, COVID-19 spread estimation, and real estate prices. 

## Figures and Tables

**Table 1:** Results of RMSE.

Well No.	DNR	WOA	PSO	GA	DE	SCA	HS
**1**	4.0578	3.5838	**2.6878**	7.8091	8.8514	5.2098	11.2956
2	2.8275	2.1646	**1.5452**	2.2508	6.9484	6.1620	12.2477
3	4.5170	2.1186	**1.5642**	3.5894	8.5141	4.7828	15.5020
4	5.0168	7.2174	**2.8998**	8.3326	13.9678	10.1031	21.9182
5	3.2376	3.0552	3.8626	**2.4013**	12.6098	7.6578	8.9531
6	4.9018	3.0601	**2.4892**	4.6272	7.7860	7.7321	19.3336
7	5.9347	**2.3293**	3.0241	4.3542	11.7616	4.4159	12.8841

**Table 2:** Results of $R^{2}$.

Well No	DNR	WOA	PSO	GA	DE	SCA	HS
**1**	0.6875	**0.8457**	0.7891	0.7450	0.7462	0.7845	0.7275
2	0.6611	**0.7753**	0.7042	0.6592	0.5444	0.6251	0.5787
3	0.6668	**0.8943**	0.8489	0.7624	0.7678	0.8037	0.7911
4	0.7103	0.8649	0.7856	**0.8802**	0.7257	0.7343	0.6554
5	0.7093	**0.9069**	0.8760	0.8482	0.8161	0.7935	0.7163
6	0.6813	**0.9049**	0.7067	0.7982	0.6741	0.7444	0.6167
7	0.6243	0.8268	**0.8639**	0.6105	0.6731	0.7354	0.8310

**Table 3:** Results of MSE.

Well No	DNR	WOA	PSO	GA	DE	SCA	HS
1	**1.2448**	3.0317	3.2014	11.0028	6.6457	3.9658	7.3400
2	0.8966	1.0981	**0.8370**	1.3937	2.7396	2.0741	4.3414
3	1.3255	0.4977	**0.4121**	0.9910	2.3315	1.2026	4.6311
4	1.5443	2.3293	**0.7931**	3.1835	4.6442	3.0516	6.5817
5	0.8842	**0.5290**	0.6313	0.6392	3.5816	1.8358	2.8121
6	1.4605	0.8906	**0.6090**	1.1794	2.1170	2.1602	5.7905
7	**1.7999**	2.7891	4.1394	8.8920	11.2291	4.1695	8.0733

**Table 4:** Results of MAE.

Well No.	DNR	WOA	PSO	GA	DE	SCA	HS
1	3.7466	3.3391	**3.3165**	7.5281	8.3595	4.9906	11.0914
2	2.5221	2.4802	**1.4894**	2.3512	6.8105	6.0657	12.0914
3	4.2634	1.8804	**1.3854**	3.2930	8.4244	4.6064	15.2934
4	4.7780	6.5083	**2.7613**	7.1260	13.4271	9.5137	21.5467
5	2.9867	2.7398	3.1507	**2.1616**	12.0793	7.3719	8.6512
6	4.7963	**2.9525**	3.1142	4.3524	7.6655	7.6302	19.1950
7	5.5770	**2.0432**	2.6755	3.9389	11.5414	4.1006	12.6019

## Data Availability

Available by corresponding author upon request.
